# Mangiferin and EGCG Compounds Fight Against Hyperlipidemia by Promoting FFA Oxidation via AMPK/PPAR*α*

**DOI:** 10.1155/ppar/7178801

**Published:** 2024-12-20

**Authors:** Yahui Xu, Jie Zhang, Ting Zhang, Minghui Zi, Qiao Zhang

**Affiliations:** Yunnan Provincial Key Laboratory of Public Health and Biosafety & School of Public Health, Kunming Medical University, Kunming, Yunnan, China

**Keywords:** *β*-oxidation, EGCG, free fatty acids, hyperlipidemia, mangiferin, phytochemicals

## Abstract

**Background:** Hyperlipidemia is a critical risk factor for obesity, diabetes, cardiovascular diseases, and other chronic diseases. Our study was to determine the effects and mechanism of mangiferin (MF) and epigallocatechin gallate (EGCG) compounds on improving hyperlipidemia in HepG2 cells.

**Methods:** HepG2 cells were treated with 0.25 mM palmitic acid (PA) and then incubated with MF (12.5, 25, and 50 *μ*M) or EGCG (25, 50, and 100 *μ*M) or MF:EGCG (0:0, 6.25:12.5, 25:50, and 50:100 *μ*M:*μ*M) for 24 h. The improvement of hyperlipidemia was verified by Oil Red O staining, changes in triglyceride (TG) and free fatty acid (FFA) levels, and the expression of lipid metabolizing proteins in western blotting.

**Results:** MF (12.5, 25, and 50 *μ*M) or EGCG (25, 50, and 100 *μ*M) markedly lowered lipid accumulations by lipid index levels. Furthermore, we found that the optimum concentration of MF and EGCG compounds was 25:50 (*μ*M:*μ*M), which significantly reduced the FFA level, TG, and total cholesterol (TC) accumulations and increased FFA uptake in HepG2 cells, and the effect was better than that of single phytochemicals. The adenosine 5⁣′-monophosphate (AMP)-activated protein kinase (AMPK) protein and its downstream proteins sirtuin 1 (SIRT1), peroxisome proliferator-activated receptor *α* (PPAR*α*), and those involved in fatty acid translocase (CD36) and carnitine palmitoyltransferase 1 (CPT1) were also markedly increased in HepG2 cells. The upregulation of protein expression was reversed when AMPK-specific inhibitor Compound C was added.

**Conclusions:** MF and EGCG (25:50 *μ*M) compounds protect against hyperlipidemia by promoting the FFA oxidation, alleviating TG and TC accumulations via the AMPK/PPAR*α* pathway in PA-treated HepG2 cells.

## 1. Introduction

Hyperlipidemia-related chronic diseases, such as cardiovascular diseases, are among the leading causes of increased morbidity and mortality worldwide [1]. Hyperlipidemia is one of the most important risk factors responsible for atherosclerosis and subsequent cardiovascular diseases [2]. Additionally, elevated triacylglycerol (TG), cholesterol (TC), and free fatty acid (FFA) levels can also lead directly to abnormal lipid accumulations, which are responsible for a less significant increase in the cardiovascular, obesity, and diabetes risk [3]. Hence, reducing blood lipids, especially TG, TC, and FFA levels reduces obesity and diabetes events in subjects both without and with known chronic diseases [4, 5].

A rich variety of lipid-lowering drugs are widely used, many of which should be used long-term and have diverse adverse side effects, including hepatorenal toxicity and neurotoxicity [6, 7]. It is still a challenge to develop more effective lipid-lowing drugs with fewer by-effects at present. It is a possible strategy to screen drugs from phytochemicals, which are currently considered to be less toxic and have numerous pharmacological functions [8]. But the single phytochemicals have limitations in dealing with complex hyperlipidemia. Currently, most studies on phytochemicals to lower lipids are generally limited to single substances or compounds of the same categories. Due to the complexity of the lipid metabolism regulatory network, the synergistic regulation of different metabolic pathways or targets may be more effective than single pathways or targets in the treatment of hyperlipidemia [9]. The combination and synergy of phytochemicals and whether they can achieve more effective lipid-lowering effects are further investigated [10].

Mangiferin (MF) is a biphenylpyrone flavonoid extracted from natural plants, which is chemically 1,3,6, 7-tetrahydroxy flavanone-C2-*β*-D-glucoside. It is widely present in many kinds of plants and Chinese herbal medicines such as *Anemarrhena asphodeloides*, *Mangifera indica* L, *Mangifera indica*, and *Mangifera persiciformis* [6, 7]. MF has plenty of beneficial biological activities, including hypolipemic, antihyperglycemic, anti-inflammatory, and antioxidant effects [11, 12]. Epigallocatechin gallate (EGCG) is the most abundant and effective dietary polyphenol extracted from tea, especially green tea, accounting for 40%–50% of tea polyphenols, which has been proven to have biological effects such as lowering blood lipids, antioxidant, hypoglycemic, anti-inflammatory, and anticancer [13–15].

Important sites of FFA removal from the blood are the liver during activity. HepG2 cells were induced to establish a high-fat model that is often used as a model to simulate hyperlipidemia in vitro. Palmitic acid (PA) is a long-chain saturated fatty acid, which is also the component with the highest content of FFAs, and has a strong ability of lipid deposition. Our previous studies found that the appropriate concentration of PA was 0.25 mM [16, 17].

Studies have found that the combination of MF and resveratrol plays a role in regulating lipid metabolism by regulating gene expression in adipose tissue [18]. EGCG and pioglitazone-cotreated PA-stimulated HepG2 cells more effectively inhibited gluconeogenesis and alleviated insulin resistance [19]. Our previous studies indicated that MF or EGCG had the effect of decreasing serum triglycerides (TGs) and FFA levels in PA-induced cells, hyperlipidemic hamsters, and rats by inhibiting lipotoxicity [20, 21]. However, the mechanism by which MF and EGCG compounds mitigated hyperlipidemia caused by lipid metabolism remains unclear. Therefore, we conducted this study to investigate the protective mechanisms of MF and EGCG compounds in the development of hyperlipidemia induced by PA in HepG2 cells based on these studies.

## 2. Materials and Methods

### 2.1. Materials

Dulbecco's Modified Eagle Medium (DMEM) was obtained from Gibco (Grand Island, NY). Fetal bovine serum (FBS) was obtained from Gibco (Grand Island, NY). MF and EGCG were purchased from Shanghai Maclean Biochemical Technology Co., Ltd (Shanghai, China). Dimethyl sulfoxide (DMSO) was purchased from Sigma–Aldrich (St. Louis, MO, United States). 3-(4, 5-Dimethylthiazol-2-yl)-2, 5-diphenyl-2Htetrazolium bromide (MTT) for cytotoxicity was obtained from MP Biomedicals (Biofroxx, Shanghai, China). TG assay kit was purchased from Jiancheng Bioengineering Institute (Nanjing, China). The total cholesterol (TC) kit was obtained from APPLYGEN (Beijing, China). FFA Assay Kit-Quantification was purchased from Abcam (Cambridge, MA, United States). The polyvinylidene difluoride (PVDF) membrane was purchased from Bio-RAD Corp (California, United States). Adenosine 5⁣′-monophosphate (AMP)-activated protein kinase (AMPK) (A12718, 1:1000), peroxisome proliferator-activated receptor *α* (PPAR*α*) (A18252, 1 : 1000), antibody against fatty acid translocase (CD36) (A1470, 1:1500), carnitine palmitoyltransferase 1 (CPT1, A5307, 1 : 1000), and *β*-actin were obtained from ABclonal Technology (Wuhan, China). Sirtuin 1(SIRT1) (A11267, 1:2000) was purchased from Hangzhou Huaan Biotechnology Co., Ltd (Hangzhou, China). Phospho-AMPK*α* (Thr172) (P-AMPK, AF3423, 1:1000) was purchased from Affinity Biosciences, OH, United States. Compound C was purchased from MedChemExpress (NJ, United States).

### 2.2. Cell Culture and Treatment

The human hepatoma (HepG2) cell line was obtained from the Chinese Academy of Sciences (Shanghai, China). The cells were maintained in DMEM containing 10% FBS and 1% antibiotic/antimycotic at 37°C in an atmosphere containing 95% air and 5% carbon dioxide (CO_2_). HepG2 cells were treated with 0.25 mM PA and then treated with MF (12.5, 25, 50 *μ*M) or EGCG (25, 50, 100 *μ*M) or MF: EGCG (0:0, 6.25:12.5, 25:50, 50:100 *μ*M:*μ*M) for 24 h.

### 2.3. Cytotoxicity

The cell cytotoxicity was tested with MTT assays. HepG2 cells were prepared into a single-cell suspension with a cell density of 1 × 10^5^cells/mL, and 100 *μ*L was inoculated in 96-well plates and cultured overnight. MF was fully dissolved with DMSO, and the concentration of the stock solution was 50 mmol/L. The EGCG was fully dissolved with sterile double steaming water, and the concentration of EGCG was prepared as 25 mmol/L. The control group was treated with 100 *μ*L complete medium, and the other treatment groups were treated with 100 *μ*L complete medium containing different concentrations (6.25, 12.5, 25, 50, 100, and 200 *μ*M) of MF or EGCG per well for 24 h. The old medium was discarded after treatment for 24 h, and 20 *μ*L MTT (5 mg/mL) solutions were added to each well and cultured in the dark for 4 h in the cell incubator. Then, the supernatant was discarded carefully; 150 *μ*L of DMSO was added, avoiding the light on a shaker for 15 min, and the light absorption value at 570 nm was measured with a microplate reader (Bio-Rad Life Medical Products Co., Ltd) [8].

### 2.4. Oil Red O Staining

HepG2 cells (1 × 10^5^/mL) were inoculated on 6-well plates with 2 mL per well and cultured in an incubator at 37°C containing 5% CO_2_. The cells were divided into a blank control group, PA group, PA+MF with different concentrations, PA+EGCG with different concentrations, and PA+MF/EGCG compound concentration group, respectively. The cells were treated for 24 h, the old medium was discarded, and the cells were washed twice with precooling PBS. The cells were fixed with 1 mL of 4% paraformaldehyde for 30 min and then stained with 1 mL of newly prepared Oil Red O (Bomeibio, Hefei, China) solution (working solution, 0.5 g Oil Red O powder dissolved in 60% isopropanol) for 30 min. The Oil Red O solution was poured off, added 1 mL PBS solution to each well, washed four times, retained PBS for the last time, and taken pictures under the inverted microscope [9].

### 2.5. Determinations of Intracellular TG, TC, and FFA

HepG2 cells (1 × 10^5^/mL) were inoculated on 6-well plates with 2 mL per well and cultured in an incubator at 37°C containing 5% CO_2_. The cells were treated for 24 h, determinations of intracellular TG, TC, and FFA. Cell protein was determined at 562 nm using the bicinchoninic acid (BCA) Protein Concentration Kit (Beyotime Biotechnology, Shanghai, China) [9]. The intracellular TG mass was quantified spectrophotometrically at 510 nm using a TG test kit (Jiancheng, Nanjing, China), and TC mass was quantified spectrophotometrically at 490 nm using a TC test kit (APPLYGEN, Beijing, China) [9]. The TG levels were calculated in millimoles per liter/gprot, and TC levels were calculated in micromole/milligram protein. The FFA content was determined by fluorescence assay using a quantitative assay kit under the condition of Ex/Em = 535/587 nm (Abcam, Cambridge, United States) [9].

### 2.6. Western Blotting Analysis

The cells (1 × 10^5^/mL) were inoculated on 6-well plates with 2 mL per well and cultured in an incubator containing 5% CO_2_ at 37°C. The cells were divided into different treatment groups and cultured for 24 h to extract cell proteins. Two hundred microliters of western and IP lysate containing 10% PMSF was added to each well and cleaved at 4°C for 30 min. The cells were collected and centrifuged in a 1.5-mL EP tube at 12,000 rpm for 20 min to obtain supernatant. The protein concentration was determined by the BCA kit. The separation gel of 8% or 10% sodium dodecyl sulfate (SDS)-polyacrylamide gel and a concentrating gel of 5% SDS-polyacrylamide gel were prepared with protein (40 or 50 ug) per well for electrophoresis. Under electrophoresis conditions, the concentrating gel is 80 V, 30 min, and the separation gel is 120 V, 1 h and 20–50 min. The protein was transferred to the polyvinylidene difluoride (PVDF) membrane by wet transfer method for 2 h. Nonspecific binding was blocked with 5% bovine serum albumin (BSA) with 0.05% Tween in triethanolamine-buffered saline solution (TBS) and incubated with primary antibodies (AMPK*α*/P-AMPK M. W 64, SIRT1 M. W 110, CPT1/CD36 M. W 88, PPAR*α* M. W 52) at 4°C for overnight [9]. Anti-rabbit alkaline phosphatase–conjugated antibody (KPL, Los Angeles, United States) was used as a secondary antibody for 1 h at 30°C. The membranes were washed three times with Tris buffered saline with tween 20 (TBST) (TBS containing 0.05% Tween). Protein bands were detected using an enhanced chemiluminescence (ECL) substrate (Beyotime Biotechnology, Shanghai, China) in a chemiluminescence imager (GE AI680, United States) and the intensities were assessed by Image J. The expression levels of various proteins were analyzed by the semiquantitative method.

### 2.7. Statistical Analysis

The Statistical Package of Social Sciences (SPSS) 26.0 software was used for all statistical analyses. Values were expressed as mean ± standard deviation. Statistical analyses were conducted by one-way ANOVA and pairwise comparisons using the LSD test, and *p* < 0.05 was considered statistically significant.

## 3. Results

### 3.1. Cell Viability

**Figure 1:**
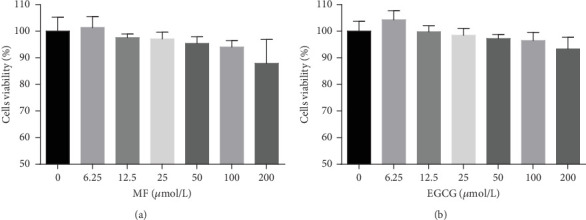
Effects of mangiferin, EGCG on HepG2 cell viability. (a) HepG2 cells were treated with different concentrations of MF (0, 6.25, 12.5, 25, 50, 100, and 200 *μ*M) for 24 h. (b) HepG2 cells were treated with different concentrations of EGCG (0, 6.25, 12.5, 25, 50, 100, and 200 *μ*M) for 24 h. Data are presented as mean ± SD (*n* = 3).

HepG2 cells were treated with 0–200 *μ*M of MF and EGCG for 24 h to examine cell viability by MTT (Figure 1). Based on the above analysis, MF concentration was safe and effective within 12.5–50 *μ*M, and EGCG was 25–100 *μ*M within HepG2 cells in the present study.

### 3.2. Mangiferin or EGCG Relief Lipid Accumulations in HepG2 Cells of TG/Lipophilic Oil Red O/FFA

**Figure 2:**
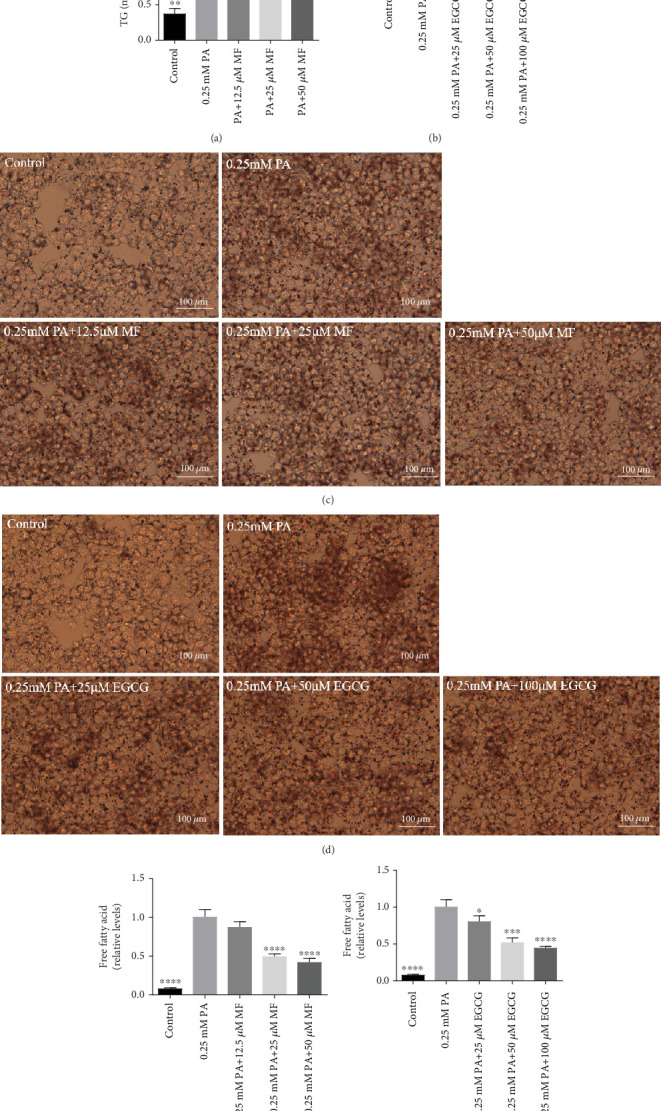
Mangiferin or EGCG relief lipid accumulations in HepG2 cells. HepG2 cells were treated with 0.25 mM of PA and 12.5, 25, and 50 *μ*M of mangiferin or 0.25 mM of PA and 25, 50, and 100 *μ*M of EGCG for 24 h. (a, b) The TG mass was quantified by using a TG test kit. (c, d) The levels of Oil Red O staining in HepG2 cells. All groups were stained with Oil Red O to observe lipid droplets at 400×. (e, f) The FFA mass was quantified by using a FFA test kit. The experiments were repeated three or four times. Data are presented as means ± SD. ⁣^∗^*p* < 0.05, ⁣^∗∗^*p* < 0.01, ⁣^∗∗∗^*p* < 0.001, and ⁣^∗∗∗∗^*p* < 0.0001 compared with the PA stimulation group.

The TG levels were increased after treatment with 0.25 mM PA, indicating that the establishment of the high fat and FFA model was due to the administration of PA. MF or EGCG treatments decreased the levels of TG (⁣^∗^*p* < 0.05 and ⁣^∗∗^*p* < 0.01; Figures 2(a) and 2(b)). Meanwhile, lipophilic Oil Red O (Figures 2(c) and 2(d)) staining showed that MF or EGCG treatments decreased hepatic steatosis through a significant decrease of FFAs (⁣^∗^*p* < 0.05, ⁣^∗∗∗^*p* < 0.001, and ⁣^∗∗∗∗^*p* < 0.0001; Figures 2(e) and 2(f)). The result indicated that the single MF or EGCG could effectively reduce the lipid accumulation in PA-treated HepG2 cells.

### 3.3. The Optimum Concentration of MF/EGCG Protected Against PA-Induced Lipotoxicity in HepG2 Cells

**Figure 3:**
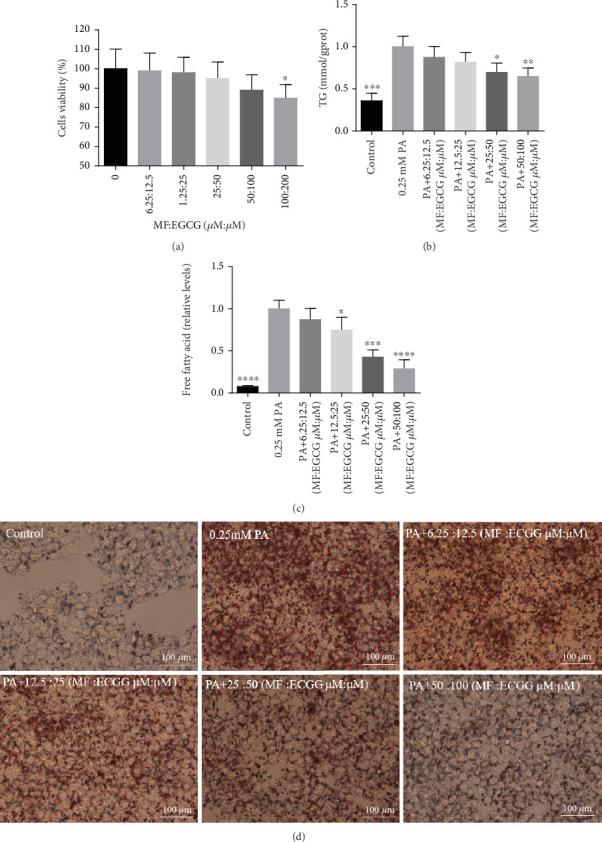
Different concentrations of MF/EGCG compound have protective effects on PA-induced lipidosis in HepG2. (a) HepG2 cells were treated with 0.25 mM of PA and MF/EGCG 6.25:12.5, 12.5:25, 25:50, and 50:100 (*μ*M:*μ*M) for 24 h. The levels of (b) TG, (c) FFAs, and (d) Oil Red O staining in HepG2 cells. All groups were stained with Oil Red O to observe lipid droplets at 400×. The experiments were repeated three or four times. Data are presented as means ± SD compared with the control group; ⁣^∗^*p* < 0.05, ⁣^∗∗^*p* < 0.01, ⁣^∗∗∗^*p* < 0.001, and ⁣^∗∗∗∗^*p* < 0.0001 compared with the PA stimulation group.

**Figure 4:**
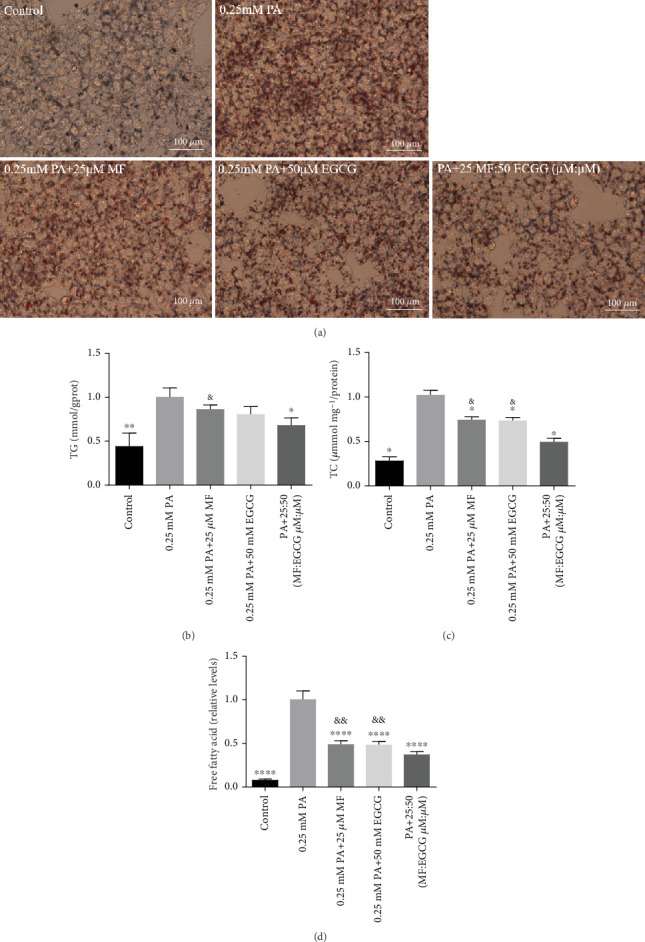
The optimum concentration of MF/EGCG protected against PA-induced lipotoxicity in HepG2 cells. HepG2 cells were treated with 0.25 mM of PA, 25 *μ*M of mangiferin, and 50 *μ*M of EGCG and MF/EGCG 25:50 (*μ*M:*μ*M) for 24 h. The levels of (a) Oil Red O, (b) TG, (c) TC, and (d) FFA staining in HepG2 cells. All groups were stained with Oil Red O to observe lipid droplets at 400×. The experiments were repeated three or four times. Data are presented as means ± SD. #*p* < 0.05, ⁣^∗^*p* < 0.05, ⁣^∗∗^*p* < 0.01, ⁣^∗∗∗^*p* < 0.001, and ⁣^∗∗∗∗^*p* < 0.0001 compared with the PA stimulation group; &*p* < 0.05 and &&*p* < 0.01 compared with the 25:50 (MF:EGCG) group.

HepG2 cells were treated with 0:0, 6.25:12.5, 12.5:25, 25:50, 50:100, and 100:200 (*μ*M:*μ*M) of MF:EGCG for 24 h to examine cell viability by MTT (*p* < 0.05, Figure 3(a)). The cell ability of HepG2 cells was markedly repressed by 100:200 (*μ*M:*μ*M) MF/EGCG (*p* < 0.05). 25:50, 50:100 (*μ*M:*μ*M) MF/EGCG treatments not only decreased the TG and FFA levels (⁣^∗^*p* < 0.05, ⁣^∗∗^*p* < 0.01, ⁣^∗∗∗^*p* < 0.001, and ⁣^∗∗∗∗^*p* < 0.0001; Figures 3(b) and 3(c)) but also relieved lipid deposition and lipid droplets (Figure 3(d)). According to the above, the MF/EGCG safe concentration with 25:50 (*μ*M:*μ*M), which was markedly lowered lipid droplets (Figure 4(a)), TG (⁣^∗^*p* < 0.05 and ⁣^∗∗^*p* < 0.01; Figure 4(b)), TC (⁣^∗^*p* < 0.05; Figure 4(c)), and FFA (⁣^∗∗∗∗^*p* < 0.0001; Figure 4(d)) compared with 25 *μ*M MF or 50 *μ*M EGCG in HepG2 cells. Furthermore, the TG, TC, and FFA levels were significantly decreased by 25:50 (*μ*M:*μ*M) MF/EGCG, compared with 25 *μ*M MF or 50 *μ*M EGCG. The above results suggest that 25:50 (*μ*M:*μ*M) MF/EGCG showed the remarkable in ameliorating lipid deposition than 25 *μ*M MF or 50 *μ*M EGCG.

### 3.4. MF/EGCG Compound Regulated the Key Proteins Involved in Lipid Metabolism in HepG2 Cells

**Figure 5:**
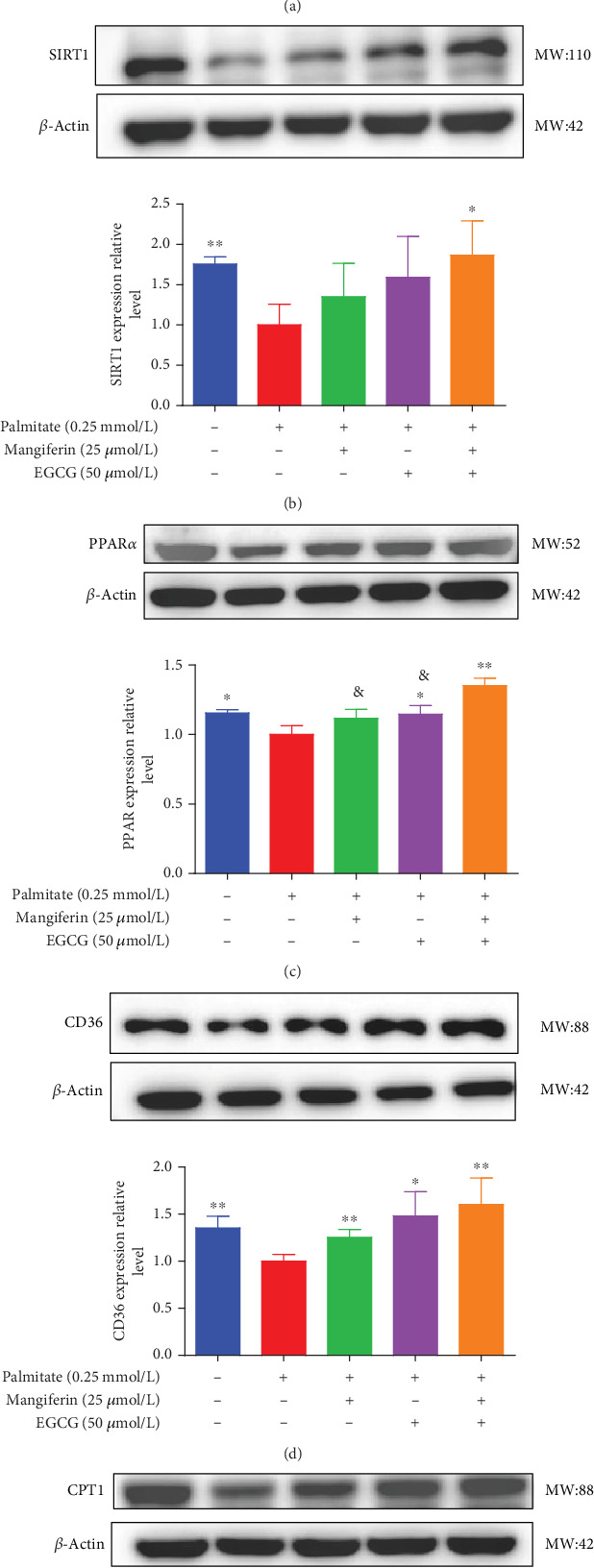
The effects of MF/EGCG compound on related proteins involved in lipid metabolism in HepG2 cells. HepG2 cells were divided into four groups: control group, PA group, PA+25 *μ*M of mangiferin, PA+50 *μ*M of EGCG, and PA + MF/EGCG 25:50 (*μ*M:*μ*M) group treated for 24 h. The proteins of (a) P-AMPK/AMPK*α*, (b) SIRT1, (c) PPAR*α*, (d) CD36, and (e) CPT1 were determined by western blot in HepG2 cells. The experiments were repeated three or four times. Data are presented as means ± SD. ⁣^∗^*p* < 0.05 and ⁣^∗∗^*p* < 0.01, compared with the PA stimulation group; &*p* < 0.05 compared with the 25:50 (MF:EGCG) group.

We examined the key proteins of lipid metabolism including AMPK, SIRT1, PPAR*α*, CD36, and CPT1 by MF/EGCG 25:50 (*μ*M:*μ*M) intervention to further verify the mechanism of MF/EGCG on lipid metabolism. The results showed that AMPK*α*/P-AMPK (⁣^∗^*p* < 0.05; Figure 5(a)), SIRT1 (⁣^∗^*p* < 0.05 and ⁣^∗∗^*p* < 0.01; Figure 5(b)), PPAR*α* (⁣^∗^*p* < 0.05 and ⁣^∗∗^*p* < 0.01; Figure 5(c)), CD36 (⁣^∗^*p* < 0.05 and ⁣^∗∗^*p* < 0.01; Figure 5(d)), and CPT1 (⁣^∗^*p* < 0.05 and ⁣^∗∗^*p* < 0.01; Figure 5(e)) were significantly increased by MF/EGCG 25:50 (*μ*M:*μ*M) in PA-treated HepG2 cells. Furthermore, the lipid metabolism–related protein expressions (P-AMPK, SIRT1, PPAR*α*, CD36, and CPT1) were increased by 25:50 (*μ*M:*μ*M) MF/EGCG, compared with 25 *μ*M MF or 50 *μ*M EGCG.

### 3.5. MF/EGCG Compound Ameliorated Lipid Metabolism via AMPK/PPAR*α* Signaling Pathway

**Figure 6:**
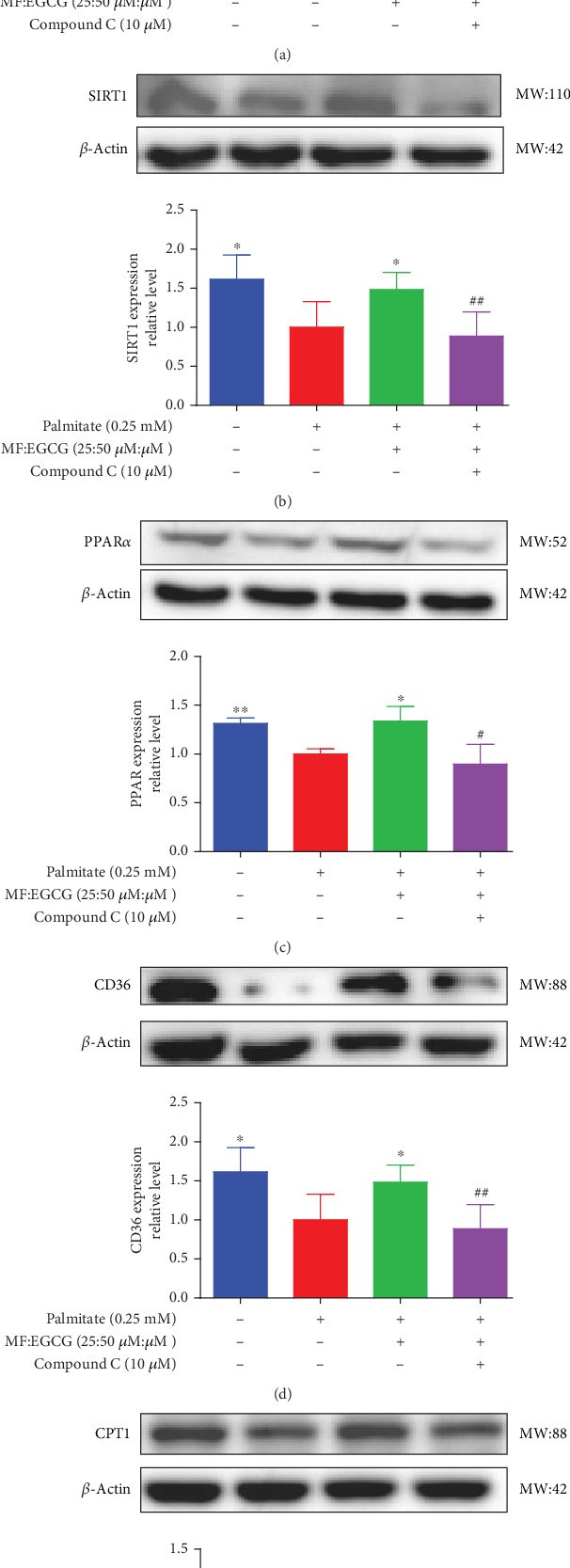
MF/EGCG compound may activate the AMPK/PPAR*α* signaling pathway to promote lipid metabolism. HepG2 cells were divided into four groups: control group, PA group, PA+MF/EGCG 25:50 (*μ*M:*μ*M) group, and PA+MF/EGCG+Compound C group (pretreated with 10 *μ*M Compound C for 1 h prior to MF/EGCG compounds treatment) for 24 h. (a) The P-AMPK/AMPK*α* expression in HepG2 cells and (b–e) the downstream proteins, including SIRT1, PPAR*α*, CD36, and CPT1expressions in HepG2 cells. The experiments were repeated three or four times. Data are presented as means ± SD. ⁣^∗^*p* < 0.05 and ⁣^∗∗^*p* < 0.01, compared with the PA stimulation group. ^#^*p* < 0.05 and ^##^*p* < 0.01, compared with the PA+MF/EGCG 25:50 (*μ*M:*μ*M) group.

To demonstrate whether the effects of EGCG on lipid metabolism are mediated by AMPK activation, a specific inhibitor of AMPK*α*, 10 mM Compound C was used. The results showed that the AMPK*α*/P-AMPK expressions were suppressed after being treated with Compound C (⁣^∗^*p* < 0.05 and #*p* < 0.05; Figure 6(a)). Meanwhile, AMPK*α* downstream proteins, including SIRT1 (⁣^∗^*p* < 0.05 and ##*p* < 0.01; Figure 6(b)), PPAR*α* (⁣^∗^*p* < 0.05, ⁣^∗∗^*p* < 0.01, and #*p* <0.05; Figure 6(c)), CD36 (⁣^∗^*p* < 0.05 and ##*p* <0.01; Figure 6(d)), and CPT1 (⁣^∗∗^*p* < 0.01 and #*p* <0.05; Figure 6(e)), were also repressed in PA-treated HepG2 cells.

## 4. Discussion

MF or EGCG shows many beneficial biological activities, including antihyperglycemic, hypolipidemic, anti-inflammatory, and antioxidant effects [10, 22]. The preventive role of MF or EGCG in dyslipidemia was assessed in several studies [7, 23]. Studies showed that MF decreased serum TG and FFA levels in hyperlipidemic hamsters and rats by inhibiting lipogenesis and promoting fatty acid oxidation [11, 12]. In addition, some studies found that supplementation with 40 mg/kg EGCG markedly suppressed atherosclerotic plaque formation and lipid accumulation in the liver and also modulated dyslipidemia [13].

The liver is one of the primary tissues of lipid metabolisms. Hyperlipidemia and hyperlipidemia-related diseases, like obesity associated with a high-fat environment, always result in metabolic disorders in the liver, such as abnormal lipid accumulation [14]. Hence, ameliorating hepatic energy metabolism is of great necessity in the treatment of hyperlipidemia and hyperlipidemia-related diseases. Although HepG2 cells are a kind of cancer cells, they displayed many genotypic features of normal human hepatocytes and were used wildly to model hepatic function in vitro in our study [15]. A saturated fatty acid, glycerides of PA occur widely in plant and animal oils and fats, which is commonly used to induce hyperlipidemia models of cells. In the previous studies, the results indicated that the appropriate and safe concentration of PA is 0.25 mM [16, 17]. Hence, in the current study, the high-fat environment was treated with HepG2 cells with 0.25 mM PA, which was in agreement with other studies [16]. The present result showed that a high-fat environment with an increase in TG, TC, and FFA levels was induced successfully by 0.25 mM PA in HepG2 cells.

Consistent with previous studies, our present results suggest that 50 *μ*M MF or 100 *μ*M EGCG obviously decreased lipid accumulations in PA-treated HepG2 cells [11, 16]. However, there are many studies that focus on single phytochemicals, and they have certain limitations in dealing with hyperlipidemia with complex etiology. Herein, MF and EGCG were scientifically compatible to determine the optimal compatibility ratio and further explore the molecular mechanism of their lipid-lowering effect in this study. Our results found that the optimum concentration of MF/EGCG (25:50 *μ*M) significantly decreased lipid droplets by Oil Red O staining and TG, TC, and FFA levels in HepG2 cells.

Then, we focused on the mechanism by which MF/EGCG (25:50 *μ*M) modulated lipid metabolism in HepG2 cells. We first evaluated the effects of MF/EGCG on the lipid metabolism of HepG2 cells in high-fat environments, which are primarily determined by the uptake and oxidation of FFA. PPAR*α* is a gene transcription related to peroxisome proliferation, which can regulate mitochondrial fatty acid oxidation in hepatocytes and is highly expressed in the liver [15, 24]. The present result was in agreement with other studies that CD36 expression was increased to enhance fatty acid uptake in the liver and PPAR*α*, and CPT1 levels were obviously decreased in the high-fat environment [17, 18, 25]. Our study demonstrated that PPAR*α*, CD36, and CPT1 were significantly increased to enhance fatty acid uptake and oxidation by MF/EGCG (25:50 *μ*M) in PA-treated HepG2 cells. The decrease degree was better than that of single 25 *μ*M MF and 50 *μ*M EGCG, and the results of combined effects with other drugs had similar conclusions. Then, how to realize the combined effect of the two, as well as the specific molecular mechanisms and signaling pathways, is worthy of further exploration.

CPT1 is the *β*-oxidation essential enzyme, which participates in lipid oxidation and fat reduction. CD36 is a receptor for several ligands, enabling their transport into cells followed by the next steps of lipids metabolisms, where it is the rate-limiting enzyme involved in *β*-oxidation and high-affinity uptake of fatty acids [19, 20, 26, 27]. The further increase in CD36 expression by MF/EGCG (25:50 *μ*M) reported that MF/EGCG might promote the utilization of fatty acid in HepG2 cells. By regulating CPT1 and CD36, MF/EGCG (25:50 *μ*M) promoted FFA *β*-oxidation to correct the lipid metabolism disorder in HepG2 cells, which was verified by the inhibition of fatty acid accumulations and the decrease of TG and TC.

AMPK is a central switch for regulating liver energy homeostasis, and activating AMPK is of great significance in the treatment of lipid metabolic disorders [21, 28]. Furthermore, previous studies mentioned that phosphorylation of AMPK*α* could activate PPAR*α* directly, and with that regard, PPAR*α* also plays a key role in the modulation of lipid metabolisms [29, 30]. As we expected, the phosphorylation of AMPK*α* was increased, which promotes its downstream proteins SIRT1, PPAR*α*, CPT1, and CD36 expressions and finally improves the lipid metabolisms by MF/EGCG (25:50 *μ*M) in PA-treated HepG2 cells.

We used Compound C to repress AMPK to further validate whether the effects of MF/EGCG (25:50 *μ*M) were mediated by activation of AMPK. Our results found that changes in most factors induced by MF/EGCG (25:50 *μ*M), including SIRT1, PPAR*α*, CPT1, and CD36, were reversed by Compound C. Furthermore, PPAR*α* expression was also obviously reduced by Compound C, which partly verified that PPAR*α* might be downstream of AMPK. These results indicated that MF/EGCG (25:50 *μ*M) exerted its beneficial metabolic effects by the activation of the AMPK/PPAR*α* pathway promoting the transport and oxidation of fatty acids and reducing lipid accumulation through regulating lipid metabolism in PA-treated HepG2 cells. However, the means by which MF/EGCG compound causes the activation of AMPK/PPAR*α* in PA exposures needs further study in vivo. Additionally, MF/EGCG could be connected and combined from a molecular structure to enhance stabilities and better bioavailability, which provides a new perspective and theoretical basis for improving and treating related diseases caused by lipid metabolism disorders in the future [31, 32].

## 5. Conclusions

Taken together, this study confirmed the novel finding that MF/EGCG (25:50 *μ*M) improved cellular lipid metabolisms in PA-treated HepG2 cells, which may be associated with the AMPK/PPAR*α* pathway. We proposed that a better understanding of MF and EGCG compounds could unseal possibilities to manipulate lipid metabolism with hyperlipidemia and hyperlipidemia-related metabolic diseases.

## Data Availability

The data that support the findings of this study are available from the corresponding author upon reasonable request.
